# Plasma-based proteomics analysis of molecular pathways in canine diabetes mellitus after astaxanthin supplementation

**DOI:** 10.1371/journal.pone.0321509

**Published:** 2025-05-07

**Authors:** Sataporn Phochantachinda, Pongsakorn Photcharatinnakorn, Duangthip Chatchaisak, Walasinee Sakcamduang, Anchana Chansawhang, Shutipen Buranasinsup, Namphung Suemanotham, Boonrat Chantong

**Affiliations:** 1 Department of Clinical Sciences and Public Health, Faculty of Veterinary Science, Mahidol University, Nakhon Pathom, Thailand; 2 Prasu Arthorn Animal Hospital, Faculty of Veterinary Science, Mahidol University, Nakhon Pathom, Thailand; 3 The Center for Veterinary Diagnosis, Faculty of Veterinary Science, Mahidol University, Nakhon Pathom, Thailand; 4 Department of Pre-Clinic and Applied Animal Science, Faculty of Veterinary Science, Mahidol University, Nakhon Pathom, Thailand; 5 Department of Pathology, Faculty of Veterinary Science, Chulalongkorn University, Bangkok, Thailand; High Point University, UNITED STATES OF AMERICA

## Abstract

The hyperglycemic state in diabetes mellitus induces oxidative stress and inflammation, contributing to diabetic tissue damage and associated complications. Astaxanthin, a potent antioxidant carotenoid, has been investigated for its potential to prevent and manage diabetes across various species; however, its effect on client-owned dogs remains poorly studied. This study explored the impact of astaxanthin supplementation on canine diabetes mellitus using a proteomics approach. A total of 18 client-owned dogs were enrolled: 6 dogs with diabetes mellitus and 12 clinically healthy dogs. The diabetic dogs received their standard treatment regimen along with daily oral supplementation of 12 mg of astaxanthin (1.5–2.4 mg/kg) for 90 days. Plasma samples were collected at the beginning and end of the study period for proteomics analysis. After astaxanthin supplementation, significant alterations in the expression of proteins associated with the complement system, coagulation cascade, JAK–STAT signaling, and protein kinase C signaling (all of which contribute to inflammation and oxidative stress) were observed. Astaxanthin exhibited potential for reducing diabetes-associated complications, such as insulin resistance, vascular dysfunction, nephropathy, and cardiac issues, even though it did not affect clinical parameters (hematology, plasma biochemistry, blood glucose, and serum fructosamine). These findings suggest that astaxanthin may be a valuable complementary therapy for managing diabetes-related complications in canines.

## Introduction

Dogs can develop a condition similar to human type 1 diabetes mellitus (DM) that is characterized by loss of pancreatic beta cells, insulin deficiency, and hyperglycemia [1]. The symptoms of canine DM include increased appetite, thirst, and urination as well as weight loss [2]. Managing canine DM is difficult because of its rapid progression and varying responses to treatment [3]. Treatments for this condition focus on controlling carbohydrate intake and insulin therapy to maintain blood glucose levels within a safe range [2]. If left untreated, chronic hyperglycemia can lead to complications such as kidney disease, heart problems, eye issues, nerve damage, and atherosclerosis [4]. These complications are thought to arise from hyperglycemia-induced oxidative stress and inflammation [5,6].

Oxidative stress is a critical factor in the pathogenesis and complications of DM. Elevated blood glucose levels accelerate oxidative stress through mechanisms such as activation of the polyol pathway, formation of advanced glycation end-products (AGEs), and overproduction of superoxide by the mitochondrial electron transport chain [7–9]. This oxidative environment leads to modifications of key proteins and activation of stress-sensitive kinases, such as c-Jun N-terminal kinase and IκB kinase, which impair insulin signaling pathways and contribute to insulin resistance [9,10]. Moreover, oxidative stress directly damages pancreatic β cells [11]. In diabetic nephropathy, oxidative stress promotes injury to glomerular and tubular cells, thus damaging the kidneys [12]. In diabetic retinopathy and neuropathy, oxidative stress induces vascular endothelial dysfunction by disrupting nitric oxide signaling and promoting inflammation, thereby leading to microvascular complications such as vision loss and nerve damage [13,14]. Oxidative stress also contributes to increased platelet activation observed in patients with DM, which may contribute to their increased atherosclerotic cardiovascular risk [15]. Furthermore, activation of the complement system plays a pathogenic role in hyperglycemia-induced renal injury [16,17]. Inflammation plays a pivotal role in the pathogenesis and progression of DM by contributing to both the development of the disease and its associated complications [9,18,19]. Chronic low-grade inflammation is a hallmark of diabetes and has been recognized as a central mechanism linking insulin resistance, β-cell dysfunction [20], metabolic disturbances [21], and disease complications, such as cardiovascular issues and diabetic neuropathy [19,22]. Therefore, addressing oxidative stress and inflammatory processes can mitigate both the onset and the progression of diabetes and its associated complications.

Recent advancements in high-throughput technologies have greatly improved the field of proteomics, offering remarkable insights into the circulating biomarkers of DM, even before clinical signs and laboratory abnormalities become evident [23]. A proteome-wide Mendelian randomization and colocalization analysis in type 2 DM illustrated the involvement of diabetes-associated proteins (e.g., AGE receptors and heat-shock proteins) in the complications of DM, thus indicating the roles of inflammation and oxidative stress in its progression [24]. A review of proteomics studies of human gestational DM revealed that a subset of proteins identified through functional analysis is involved in the regulation of insulin or indirect signaling pathways [25]. These pathways encompass the progression of complement and coagulation cascades, ECM–receptor interactions, focal adhesion, the PI3K–Akt signaling pathway, platelet activation, and the peroxisome proliferator–activated receptor signaling pathway [26]. In our previous proteomics analysis of diabetic dogs supplemented with curcuminoids, we discovered alterations in the expression of alpha-2-HS-glycoprotein, transthyretin, apolipoprotein A-I, and apolipoprotein A-IV [27], suggesting that curcuminoids may enhance insulin sensitivity and mitigate cardiovascular complications in dogs.

Given the significant role of oxidative stress and inflammation in the various complications of DM, extensive research has been conducted on the antioxidant effects of multiple substances, including natural antioxidants [28]. Astaxanthin, a well-studied carotenoid, has demonstrated beneficial impacts in both preventing and treating diabetes in many species owing to its antioxidant and anti-inflammatory properties [29].

Astaxanthin supplementation has gained considerable attention across various fields, including human medicine and veterinary science, because of its therapeutic potential for managing oxidative stress–related conditions, such as DM and cardiovascular complications [30–32]. In humans, astaxanthin has yielded significant antioxidant and anti-inflammatory effects, which help modulate metabolic pathways by improving insulin sensitivity and glycemic control [30,31]. Furthermore, astaxanthin enhances insulin sensitivity, promotes glucose uptake, reduces liver lipid synthesis, and provides protective effects against diabetic complications, such as retinopathy, nephropathy, and neuropathy [29,33]. In veterinary science, astaxanthin is being increasingly used in companion animals, as it has been shown to improve antioxidant function, liver function, and lipid metabolism in dogs, particularly in those with obesity [34]. Moreover, astaxanthin supplementation has enhanced immune responses in dogs, as indicated by increased lymphocyte proliferation, natural killer cell activity, and elevated immunoglobulin levels [35]. In exercise-conditioned dogs, astaxanthin improved nutritional profiles and supported glycogen and protein synthesis [36]. Furthermore, it has been shown to increase ATP synthesis and mitochondrial function in both young and geriatric Beagle dogs, with more pronounced effects observed in older animals [37].

These studies suggest that astaxanthin holds promise as a supplement for preventing and managing diabetes and its associated complications in dogs. However, despite its promising benefits across species, the molecular mechanisms underlying the effect of astaxanthin on canine diabetes remain unexplored, particularly regarding the proteomics profiles of diabetic dogs treated with astaxanthin and the effects of this agent on proteins associated with diabetic complications. Therefore, addressing this gap may provide crucial insights into the therapeutic potential of astaxanthin for canine diabetes. By delving deeper into the molecular mechanisms at play in this setting, we can pave the way for developing more precise and effective treatments for diabetes.

## Materials and methods

### Animals

A total of 18 dogs owned by clients of the Prasuarthorn Hospital, Faculty of Veterinary Science, Mahidol University, Thailand, were enrolled in the study. This study was conducted in strict accordance with the recommendations of the Guide for the Care and Use of Laboratory Animals of the National Institutes of Health. The study protocol was approved by the Committee on the Care and Use of Laboratory Animals in the Faculty of Veterinary Science, Mahidol University, Thailand (approval number: MUVS-2020-04-10). The owners were informed about the study and provided written consent prior to enrollment. The dogs were divided into two groups: dogs with DM (n = 6) and clinically healthy dogs (n = 12). DM was identified in dogs based on the presence of fasting hyperglycemia, high blood fructosamine levels, and glucosuria. The inclusion criteria for DM included dogs of any breed, age, or sex that maintained a consistently stable blood glucose level for at least 3 months. To reduce potential confounding factors, dogs with the following complications were excluded: diabetic ketoacidosis, acromegaly, exocrine pancreatic insufficiency, or neoplasia.

The dogs with DM (age, 5–11 years; body weight, 6.9 ± 1.78 kg) received their regular treatment plus daily oral supplementation with 12 mg of astaxanthin (Astalif, ALGALIF^®^, Madre Labs, LLC, CA; Lot UC210456) for 90 days. This study adopted a dose of astaxanthin (1.5–2.4 mg/kg) that is similar to that used in previous studies with dogs [35,37,38]. The astaxanthin capsule was administered either with a meal or immediately after a meal. The clinically healthy group included adult dogs of any breed or sex that were age-matched to dogs in the diabetic group and exhibited normal vital signs and blood test results for a cross-sectional baseline assessment of well-being.

Blood samples (5–9 mL) were collected from the cephalic or saphenous vein to evaluate clinical parameters such as hematology, biochemistry, blood glucose, and serum fructosamine. Urine samples were obtained via midstream voiding (to minimize stress) and stored at 4°C for urinalysis within 4 h. Clinical parameters were evaluated among the dogs with DM every 6 weeks from day 0 (DM D0) to day 90 (DM D90), whereas in the clinically healthy group, these were only assessed on day 0. Proteomics markers were measured in the DM group before and after supplementation with astaxanthin (days 0 and 90). The methods, procedures, and storage protocols used for analyzing blood and urine samples were in agreement with those described in our previous study [27].

### In-solution digestion

Protein purification was performed using the clean-up kit from GE Healthcare (USA). Subsequently, the protein pellets were dissolved in 8 M urea, and their concentrations were determined using the Bradford assay (Bio-Rad protein assay, Bio-Rad Laboratory, CA). Each sample (containing 30 μg of protein) underwent reduction with 100 mM dithiothreitol in 100 mM TEAB at 37°C for 30 min. An alkylating buffer (100 mM iodoacetamide in 100 mM TEAB) was then added, and the sample was incubated in the dark for 30 min at 25°C. After quenching with the reduction buffer for 15 min at 25°C, the sample was digested using trypsin gold (mass spectrometry grade; Promega, USA) for 16 h at 37°C. The purified peptide was dried using a nitrogen evaporator and stored at −80°C. The sample was then reconstituted in 0.1% formic acid. Finally, the peptide concentration was quantified using a NanoDrop 1,000 instrument (Thermo Fisher Scientific, Bremen, Germany).

### Nano-LC-MS/MS analysis, data processing, and statistical analysis

Peptides were analyzed using a nano-liquid chromatograph (Dionex Ultimate 3,000, RSLCnano System, Thermo Scientific) coupled with a quadrupole ion trap mass spectrometer (Model Q-ToF Compact, Bruker, Germany). A C18 column was used to enrich and separate 1 µg of peptides. Elution was performed using a linear gradient with a constant flow rate (300 nL/min) at 60°C, with two mobile phases: A) 0.1% formic acid in water, and B) 0.08% formic acid in 80% acetonitrile. MS acquisition was performed in positive ionization mode with a specific mass range of 150–2,200 m/z.

During the discovery phase, the raw MS data were processed using MaxQuant software version 1.6.2.10 (https://www.maxquant.org), along with its integrated search engine, Andromeda, for protein identification. The default settings used the *Canis lupus familiaris* database from www.uniprot.org, and the analysis was set to 1 for label-free approach. The oxidation of methionine and acetylation of the N-terminus were set as variable modifications, while the carbamidomethyl modification of cysteine was set as a fixed modification. The false discovery rate was set to 1% at the peptide level. The TOF MS/MS match tolerance was set at 0.5 Da for label-free quantification. Subsequently, label-free quantification was performed and imported into Perseus software version 1.6.8.0 (https://www.maxquant.org) for statistical analysis of differential expression using 2-sample tests, with statistical significance set at *P*-value of <0.05. The mass spectrometry proteomics data have been deposited in the ProteomeXchange Consortium via the PRIDE [39] partner repository (dataset identifier: PXD055138).

### Pathway enrichment analysis

In comparative proteomics, proteins are considered differentially expressed (either upregulated or downregulated) when their concentration changes yield an analysis of variance (ANOVA) *P*-value of <0.05. The proteins of interest were analyzed using various tools, such as PANTHER, Search Tool for the Retrieval of Interacting Genes (STRING), and KEGG (accessed on 27 February, 2024; www.string-db.org, http://pantherdb.org). The STRING database version 12.0 (https://string-db.org/, accessed February 27, 2024) [40] was used to identify relationships among proteins that underwent significant changes after reprogramming. A list of altered proteins from each time point was uploaded, with *Canis lupus* selected as the organism of origin.

### Gene Ontology (GO) annotation and pathway enrichment analysis

Proteins were categorized through GO annotation into three primary domains: biological processes, cellular components, and molecular functions. This classification aids in understanding the biological roles and processes associated with the differentially expressed proteins. Their interactions within a protein network were evaluated using the STRING database (https://string-db.org). This resource provides data on both established and predicted protein interactions, thereby helping the investigation of protein networks and their potential functional interrelations. Pathways with false discovery rates below 0.05 were deemed statistically significant. Protein functions were identified through the UniProt database (www.uniprot.org).

### Post-processing of the data

The intensities were processed to describe the proteins in terms of their relative abundance. The intensity of each identified protein was calculated as a percentage of the total by calculating the fraction of each intensity in the whole sample, as follows: (intensity of the protein or peptide/sum of the intensities of all the proteins or peptides in the sample) × 100. This generated percentage values for the identified proteins.

### Statistical analysis

Quantitative variables were summarized as the mean ± standard deviation (SD) and 95% confidence intervals, with normality assessed using the Shapiro–Wilk test. Statistical analyses of quantitative variables were performed using SPSS Statistics for Windows, version 18.0 (SPSS Inc., Chicago, IL, USA). A one-way ANOVA was performed to identify significant differences among the healthy adult, DM D0, and DM D90 groups. A post hoc analysis using one-way ANOVA followed by Tukey’s multiple comparisons test was used to assess group-specific differences. Statistical calculations were performed using GraphPad Prism version 5.0 for Windows (GraphPad Software, Boston, MA, USA; www.graphpad.com), with *P* < 0.05 indicating statistical significance.

## Results

### Population characteristics and biochemical analysis of dogs in a proteomics study

The population characteristics of diabetic dogs in the proteomics study are listed in S1 Table. The hematological and blood chemistry findings (glucose, BUN, creatinine, ALT, ALP, AST, albumin, globulin, total protein, calcium, and total bilirubin) of diabetic dogs did not significantly differ between before and after astaxanthin supplementation (*P* > 0.05), as shown in S2 Table.

### Venn diagram analysis

**Fig 1:**
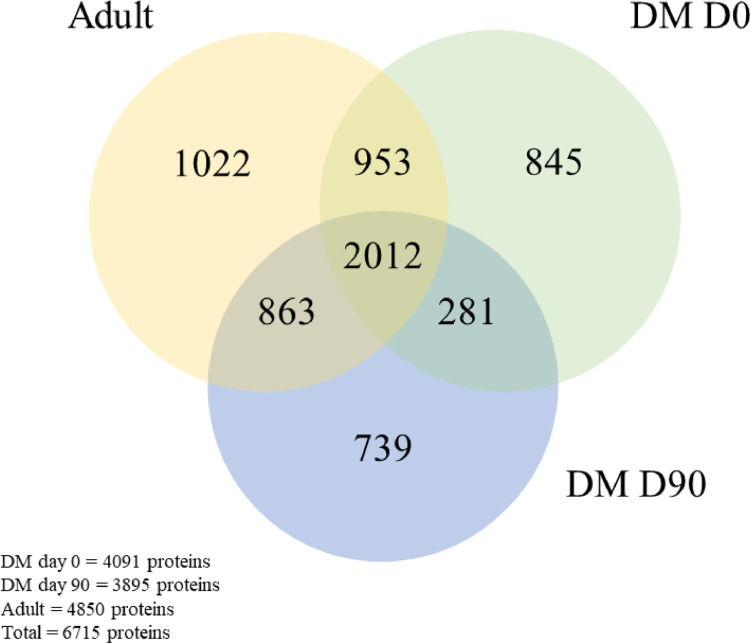
Venn diagram of protein detection (*Canis* spp.).

All plasma samples were combined in a 2:1 ratio within each group. These pooled samples were then processed to determine differences in protein expression using a proteomics approach. The raw data acquired from mass spectrometry were analyzed using the MaxQuant software to determine the quantity of proteins. Fig 1 illustrates the distinct and shared proteome profiles of the three groups. Across all groups, 2012 out of 6,715 canine proteins (29.96%) were commonly expressed. There were 2,965 (44.15%) common proteins between the DM D0 and control groups, as well as 2,293 (34.15%) common proteins between the DM D0 and DM D90 groups.

### Principal component analysis

**Fig 2:**
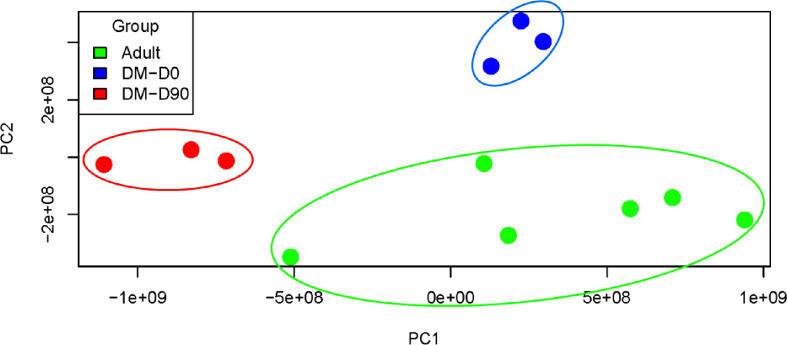
Principal component analysis (PCA). Healthy adult dogs (green dots) and diabetic dogs before (DM D0, blue dots) and after (DM D90, red dots) astaxanthin supplementation.

A principal component analysis was performed to assess the differences in metabolic or physiological profiles among healthy adult dogs (green dots), DM D0 (blue dots), and DM D9 (red dots). A biplot (Fig 2) was used to illustrate the proteome profiles of 6,715 proteins. The green dots (control group) formed a distinct cluster that was distinctly separated from the blue and red dots (both diabetic groups) along the PC1 axis, indicating a clear difference in the metabolic/physiological profile between healthy and diabetic dogs. Interestingly, after astaxanthin supplementation in diabetic dogs, the red dots in both PC1 and PC2 dimensions shifted toward the green cluster, suggesting that astaxanthin treatment induced changes in the metabolic profile of diabetic dogs, thereby partially restoring it toward a healthier state.

#### Volcano plot.

The volcano plots represent the differences in protein expression from the shotgun proteomics dataset. Substantially distinct proteins were identified as those with a fold-change of >2, implying that the log2 ratio was either >1 or <−1. As statistical significance was set at *P-*value of <0.05, the negative logarithm of the *P*-value was >1.3.

**Fig 3:**
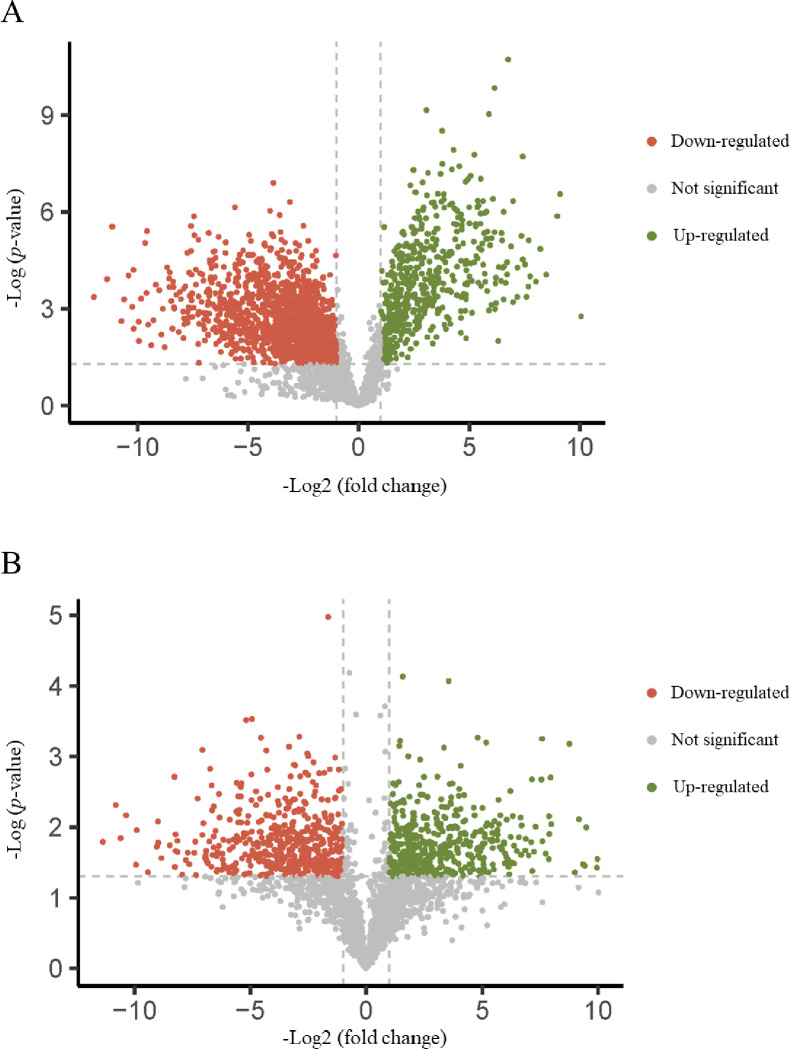
Volcano plots. (A) Diabetic dogs before astaxanthin supplementation (DM D0) vs. healthy adult dogs; (B) DM D0 vs. diabetic dogs after astaxanthin supplementation (DM D90). Each dot represents a protein. The red dots indicate significantly downregulated proteins (*P* < 0.05, fold-changes < 0.5). The green dots indicate significantly upregulated proteins (*P* < 0.05, fold-changes > 2).

Fig 3A depicts the proteins that exhibited significant differences in expression between diabetic dogs (DM D0) and healthy adult dogs, with a total of 2,968 differentially expressed proteins identified. Specifically, 499 and 1,374 proteins were significantly upregulated and downregulated, respectively, in diabetic dogs. Fig 3B compares the proteomics profiles at DM D0 and DM D90, with a total of 2,292 differentially expressed proteins identified. Specifically, 428 and 509 proteins were significantly upregulated and downregulated, respectively, after astaxanthin supplementation in diabetic dogs.

### GO and pathway enrichment analyses of proteins

**Fig 4:**
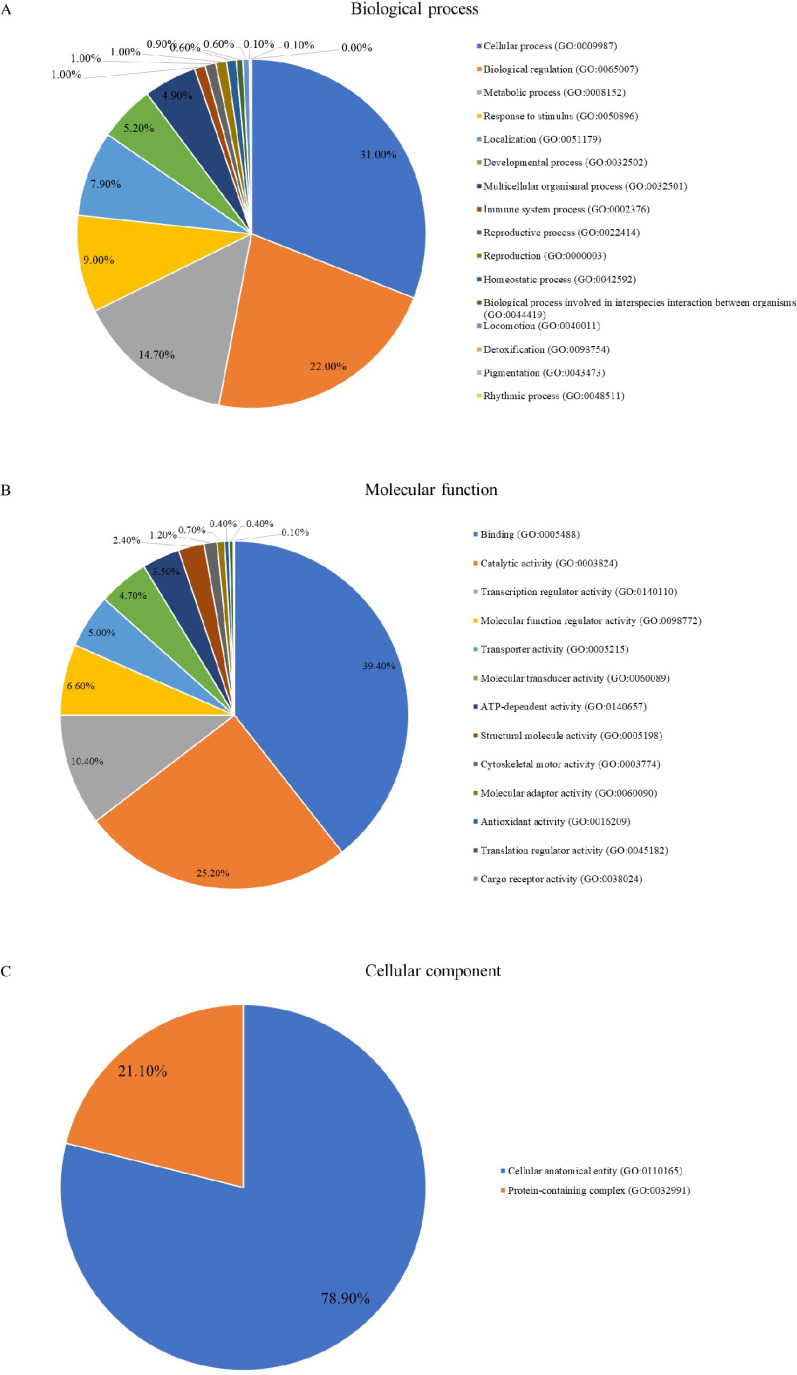
Gene Ontology annotation. Gene Ontology annotation of differentially expressed proteins pertaining to **(A)** biological processes, **(B)** molecular function, and **(C)** cellular components between diabetic dogs before (DM D0) and after (DM D90) astaxanthin supplementation and healthy adult dogs.

A pathway enrichment analysis was performed to map the proteins onto GO databases via PANTHER. Fig 4 highlights the effects of diabetes and astaxanthin supplementation on biological processes (Fig 4A), molecular functions (Fig 4B), and cellular components (Fig 4C) in diabetic dogs. The 2,549 differentially expressed proteins were grouped into three main GO categories: biological process, molecular functions, and cellular components. In the biological process category, proteins were mapped to cellular processes (n = 1,130), biological regulation (n = 804), and metabolic processes (n = 536). In the molecular function category, proteins were involved in binding (n = 777), catalytic activity (n = 498), and molecular transcription regulation (n = 206). Regarding cellular components, 1,591 proteins were associated with cellular anatomical entities, including cellular structures and membrane-enclosed cellular compartments. Therefore, diabetes and astaxanthin affect cellular processes, biological regulation, and metabolism.

### Proteins of interest and subcellular distribution of differentially expressed proteins

**Fig 5:**
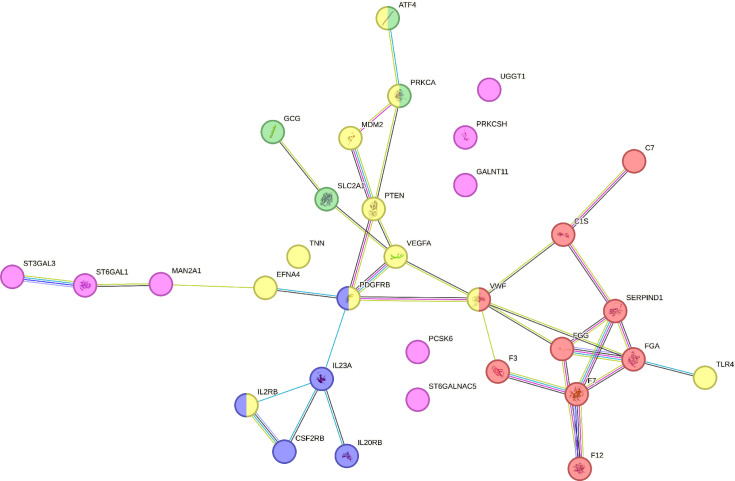
STRINGS network analysis of protein–protein interactions. Analysis of differentially regulated proteins in diabetic dogs (DM D0) vs. healthy controls and diabetic dogs after astaxanthin supplementation (DM D90) (red: complement and coagulation cascade; blue: JAK–STAT signaling pathway; green: insulin secretion; yellow: PI3K–Akt signaling pathway; pink: glycoprotein metabolic process). Each node represents a unique protein, whereas the edges connecting the nodes indicate known or predicted interactions.

To gain a deeper understanding of the complexity of these proteins, a network analysis of protein–protein interactions was performed. The STRING database was used to compare proteomics changes among the three groups. Fig 5 reveals the biological pathways in which the differentially expressed proteins were involved. In DM D0, proteins involved in (i) the complement and coagulation pathways (red dots), (ii) JAK–STAT signaling (blue dots), and (iii) insulin secretion (green dots) were considerably upregulated; whereas proteins involved in (i) the PI3K–Akt signaling pathway (yellow dots) and (ii) the glycoprotein metabolic process (pink dots) were significantly downregulated compared with the controls and DM D90. The complement pathway, coagulation pathway, and JAK–STAT signaling play a role in immune functions and inflammation [41–45].

### Protein expression

**Fig 6:**
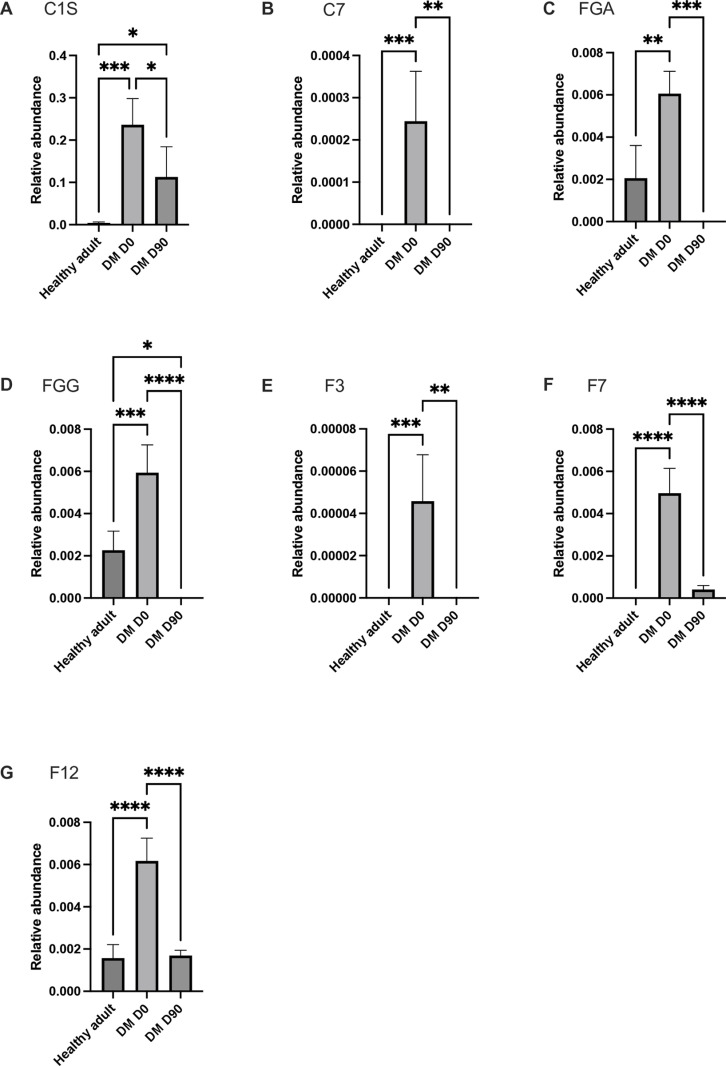
Bar chart comparing the relative intensity of proteins in the complement and coagulation cascades across the three groups. (A) C1S, (B) C7, (C) FGA, (D) FGG, (E) F3, (F) F7, and (G) F12. *Statistically significant at *P* < 0.05, **statistically significant at *P* < 0.01, ***statistically significant at *P* < 0.001, ****statistically significant at *P* < 0.0001.

**Fig 7:**
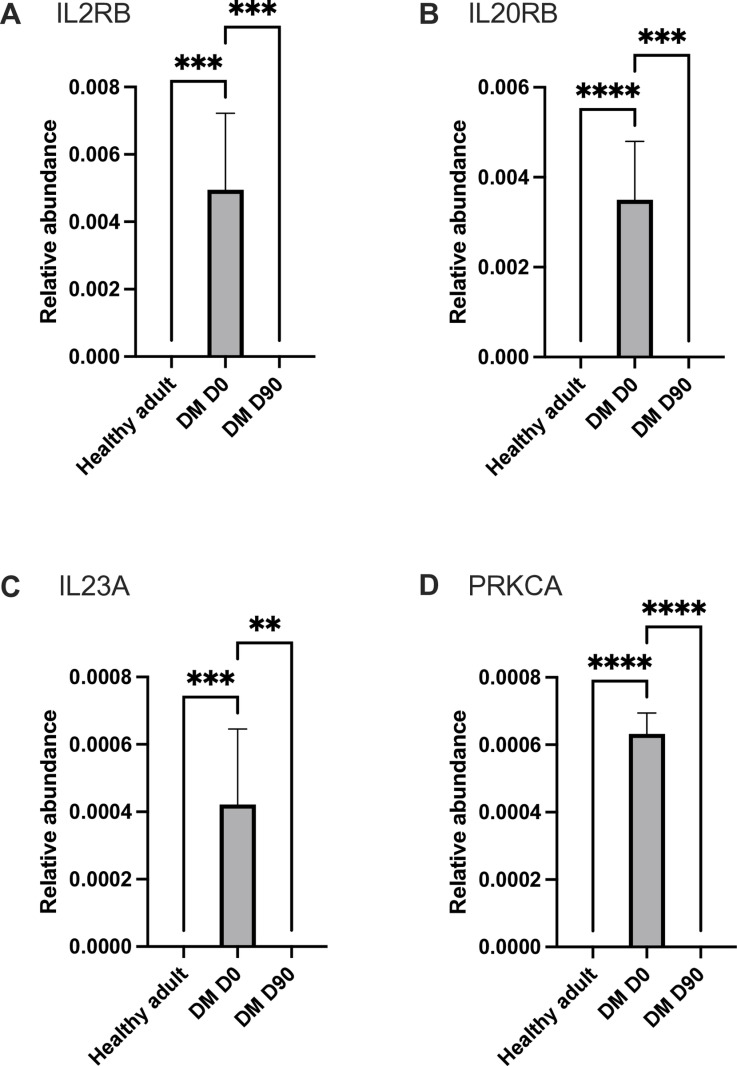
Bar chart comparing the relative intensity of proteins in JAK–STAT signaling pathway and insulin secretion across the three groups. (A) IL2RB, (B) IL20RB, (C) IL23A, and (D) PRKCA **Statistically significant at *P* < 0.01, ***statistically significant at *P* < 0.001, ****statistically significant at *P* < 0.0001.

**Fig 8:**
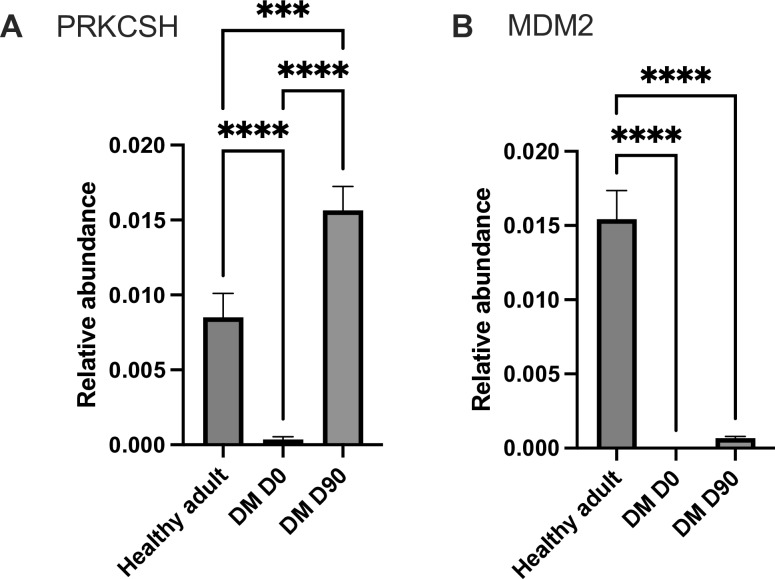
Bar chart comparing the relative intensity of proteins in PI3K–Akt signaling pathway and glycoprotein metabolic process across the three groups. (A) PRKCSH and (B) MDM2 ***Statistically significant at *P* < 0.001, ****statistically significant at *P* < 0.0001.

The proteomics analysis identified 13 proteins of interest (11 upregulated and 2 downregulated proteins), thus demonstrating differential protein expression in the plasma of the DM D0 group compared with the control and DM D90 groups. Proteins involved in the complement and coagulation cascade were notably upregulated in DM D0 (Fig 6): complement C1S (*P* < 0.001), complement C7 (*P* < 0.001), fibrinogen alpha chain (*P* < 0.001), fibrinogen gamma chain (*P* < 0.0001), factor III (*P* < 0.001), factor VII (*P* < 0.0001), and factor XII (*P* < 0.0001). Proteins involved in the JAK–STAT signaling pathway, such as interleukin (IL)-2 receptor subunit beta (*P* < 0.001) and IL-23 subunit alpha (*P* < 0.001), were also significantly upregulated (Fig 7). Protein kinase C alpha (*P* < 0.0001), which is involved in the insulin secretion pathway, was significantly upregulated. Conversely, the E3 ubiquitin-protein ligase Mdm2 (*P* < 0.0001), which is involved in the PI3K–Akt signaling pathway, was significantly downregulated. Furthermore, protein kinase C substrate 80K-H (*P* < 0.0001), which is involved in glycoprotein metabolic processes and calcium ion binding, was downregulated, indicating potential disruptions in kinase activities and metabolic processes (Fig 8). Astaxanthin treatment differentially affected proteins involved in the complement and coagulation cascades and JAK–STAT signaling pathways, with varying degrees of normalization in signal intensity. The mean intensity ± SD and 95% confidence intervals of the proteins of interest are presented in S3 Table.

## Discussion

This study aimed to elucidate the molecular mechanisms underlying canine diabetes and the potential therapeutic effects of astaxanthin. To the best of our knowledge, this study is the first to specifically document the effects of oral astaxanthin supplementation in client-owned diabetic dogs. this is the first study documenting the effects of oral astaxanthin therapy in client-owned diabetic dogs. Our results showed that blood glucose and hematological parameters remained unchanged after 90 days of astaxanthin administration. Moreover, the results of liver and kidney blood tests remained unchanged, indicating the safety of oral astaxanthin. Interestingly, proteome changes were clearly observed in astaxanthin-treated dogs, particularly involving proteins related to oxidative stress, inflammation, and insulin signaling pathways, including the complement and coagulation cascade, JAK–STAT signaling, and protein kinase C signaling pathways.

The present findings indicate that diabetes disrupts proteins that are essential for cellular functions, particularly those involved in metabolism and immune responses. Astaxanthin supplementation appeared to mitigate these disruptions through its antioxidant and anti-inflammatory properties, thus potentially improving cellular health in diabetic dogs [9,18,46–48]. The comprehensive use of analytical methods, including Venn diagrams, principal component analysis (PCA), and volcano plots, provided robust insights into these effects. Notably, the PCA demonstrated astaxanthin’s ability to partially restore a healthy metabolic profile in diabetic dogs. The GO enrichment analysis further highlighted the molecular and cellular effects of diabetes and astaxanthin, revealing a significant change in metabolic pathways and immune responses, with alterations in protein binding and catalytic activity. These results align with known metabolic dysregulation occurring in diabetes, suggesting that immune modulation and changes in enzymatic activity contribute to disease progression [49–54]. The therapeutic effect of astaxanthin appears to involve the modulation of these pathways, reducing oxidative stress and inflammation, and normalizing metabolic and cellular functions [29,33,55,56].

Network analysis highlighted significant disruptions in key pathways in diabetic dogs, including the upregulation of proteins in the complement and coagulation cascades, which reflected a link between increased inflammation, procoagulant states, and cardiovascular complications [16,41–43,57–63]. Similarly, proteins in the JAK–STAT pathway were modulated, indicating immune dysregulation that is characteristic of early-stage diabetes [44,45,64,65]. The upregulation of proteins involved in insulin secretion suggested compensatory responses or dysfunction due to elevated glucose levels. In contrast, the downregulation of proteins involved in the PI3K–Akt and glycoprotein metabolism pathways indicated impaired cell survival, metabolism, and glycosylation processes [66–70]. Astaxanthin treatment appeared to normalize protein expression in these pathways, highlighting its potential to counteract diabetes-induced molecular changes.

The GO enrichment and protein–protein interaction analyses revealed significant alterations in key pathways related to the complement system, coagulation cascade, and JAK–STAT signaling, particularly protein kinase C. The complement and coagulation systems, which are essential for immune response and hemostasis, contribute to diabetic complications, including insulin resistance, β-cell dysfunction, nephropathy, and retinopathy [16,42,57–60]. Hypercoagulability and impaired fibrinolysis that occur in diabetes increase the risk of atherosclerosis, thrombosis, and poor wound healing [61–63,71–73]. In this study, diabetic dogs exhibited elevated levels of key proteins in the complement and coagulation cascades, including complement C1, complement C7, fibrinogen alpha/gamma chains, and coagulation factors III, VII, and XII, which are associated with inflammation, insulin resistance, and increased cardiovascular risk. Complement C1s are associated with insulin resistance and diabetic retinopathy, whereas complement C7 has been associated with the early stages of diabetic kidney disease [74–76]. Moreover, elevated fibrinogen levels contribute to atherosclerosis and chronic inflammation in type 2 diabetes [77, 78]. Furthermore, diabetes is characterized by a hypercoagulable state, with increased levels of clotting factors exacerbating the risk of thrombosis and vascular complications [61–63,79,80]. Astaxanthin supplementation significantly downregulated these proteins, suggesting its potential role in mitigating inflammation and coagulopathy associated with diabetes.

Elevated serum levels of inflammatory mediators involved in JAK–STAT signaling have been associated with immune dysregulation in diabetes, thus contributing to insulin resistance, β-cell dysfunction, and endothelial impairment [64,65]. In this study, diabetic dogs exhibited significantly increased plasma levels of IL-2 receptor beta subunit, IL-20 receptor beta subunit, and IL-23 receptor alpha subunit, all of which were reduced after astaxanthin supplementation. Increased levels of soluble IL-2 receptors have been reported previously in patients with type 1 diabetes with vascular complications, further supporting the link between immune dysfunction and diabetes progression [64,65]. Moreover, IL-20, which is a pro-inflammatory cytokine that has been implicated in renal disease, is upregulated in diabetic animal models and human patients, although its direct association with diabetes remains unclear [81]. The involvement of the IL-23 receptor in chronic inflammatory conditions and T-cell differentiation further suggests a potential role in autoimmune diabetes [82,83]. The reduction in these inflammatory markers occurring after astaxanthin treatment highlights its potential to modulate immune signaling pathways and mitigate diabetes-associated inflammation.

Protein kinase C alpha (PKC-α), which is a key regulator of cellular processes and a component of the JAK–STAT pathway, plays a critical role in the pathophysiology of diabetes by promoting insulin resistance, vascular dysfunction, nephropathy, and cardiovascular complications [84–86]. In this study, diabetic dogs exhibited significantly elevated PKC-α levels compared with healthy controls, which were effectively normalized after astaxanthin supplementation, suggesting a potential therapeutic role in mitigating diabetes-related complications. Furthermore, the 80K-H protein (PRKCSH), which is involved in PKC regulation, insulin signaling, β-cell function, and GLUT4 vesicle trafficking,[87,88] was reduced in diabetic dogs but significantly increased after astaxanthin treatment, further supporting its role in the restoration of metabolic balance. Conversely, MDM2, an E3 ubiquitin ligase that modulates JAK–STAT and PI3K–Akt signaling and plays a protective role in diabetic kidney disease through NRF-2 regulation [89–92], was significantly downregulated in diabetic dogs, with no observed improvement detected after astaxanthin supplementation. These findings suggest that although astaxanthin may attenuate PKC-α–mediated complications and restore 80K-H levels, its effects on MDM2 and related pathways require further investigation.

Astaxanthin supplementation induced proteomics changes through its multifaceted mechanisms, targeting oxidative stress, inflammation, and insulin signaling, which are key drivers of diabetic complications. Its potent antioxidant properties mitigate oxidative damage by scavenging reactive oxygen species (ROS) and enhancing endogenous antioxidant enzymes, normalizing proteins involved in the complement and coagulation cascades, such as complement and fibrinogen, which are implicated in vascular dysfunction [33]. Astaxanthin also exerts anti-inflammatory effects by inhibiting the NF-κB pathway, reducing pro-inflammatory cytokines (i.e., IL-6 and TNF-α) and downregulating the JAK–STAT signaling pathway, which is associated with immune dysregulation [29,93,94]. Moreover, it may improve insulin sensitivity by modulating the PI3K–Akt pathway and normalizing insulin-related proteins, such as adiponectin receptor 1 and PKC-α [33,95]. These effects extend to reducing the hyperactivation of complement and coagulation cascades, influencing glycoprotein metabolism by upregulating PRKCSH (80K-H), and enhancing mitochondrial function by improving ATP synthesis and reducing ROS production [96,97]. The observed proteome alterations likely result from astaxanthin’s potential to simultaneously control oxidative stress, inflammation, and insulin signaling, exerting synergistic effects across multiple pathways. These findings highlight the potential application of astaxanthin as a supplement to prevent diabetes complications.

The present study suggests that astaxanthin may reduce diabetes complications by targeting oxidative stress and inflammatory molecular pathways. The proteomics alterations discovered in this study reflect early-stage molecular modifications, although they did not lead to clinical improvements, for several reasons. Diabetes is a multifactorial disease that involves chronic hyperglycemia, oxidative stress, and inflammation, making it challenging to directly correlate molecular changes with clinical outcomes. Although astaxanthin may modulate specific pathways, the complexity of diabetes may limit the immediate clinical impact of these molecular improvements, which blood glucose and fructosamine levels may not detect. Another contributing factor could be the variability in baseline conditions among diabetic dogs, including differences in insulin therapy, diet, and the severity of complications. Variations in meal composition, timing, and glycemic index could impact glucose metabolism, oxidative stress, and inflammation, thus highlighting the need for dietary standardization [98,99]. Similarly, inconsistent insulin types, dosages, and schedules may obscure astaxanthin’s effects, emphasizing the importance of protocol standardization [100]. The progression and severity of diabetes also vary among individuals, which affects baseline oxidative stress and inflammation. Stratifying subjects according to disease stage may help isolate astaxanthin’s effects [101,102]. In addition, owner compliance with feeding, medication, and environmental factors introduces variability, which may be mitigated through detailed instructions and compliance monitoring. Addressing these factors in future is crucial for accurately evaluating the therapeutic potential of astaxanthin.

The absence of a validation step for protein expression was a key limitation of this study, which may have enhanced data reliability. Although traditional methods, such as western blotting and ELISA, are commonly used, they face challenges, including limited antibody specificity and throughput [103,104]. In this study, we used label-free proteomics to show that the intensity of the peptide ion signals detected by mass spectrometry correlated with the relative abundance of the corresponding proteins, thus providing robust, reproducible, and semi-quantitative data by analyzing peptide ion intensities [105,106]. This approach is widely accepted for large-scale proteomics studies and is often sufficient in exploratory research because of advancements in bioinformatics and normalization techniques [104,107–109]. Although future studies could include complementary validation methods for specific biomarkers, the MS-based methodology used here provides reliable insights into the molecular mechanisms underlying canine diabetes.

This study provides preliminary insights into the molecular effects of astaxanthin in canine DM. However, further research is needed to validate and expand these findings. Larger and more diverse cohorts are required to confirm the proteomics changes observed here, and a longer study duration may clarify whether these molecular alterations translate into meaningful clinical benefits, such as improved glycemic control and reduced diabetic complications. Validation of identified proteins as biomarkers is essential, with correlations to clinical metrics, such as blood glucose, insulin sensitivity, and inflammatory markers. Mechanistic studies should investigate the direct effects of astaxanthin on key pathways, particularly the complement system, the coagulation cascade, and JAK–STAT and protein kinase C signaling. Comparative studies assessing astaxanthin in combination with other antioxidants or anti-inflammatory agents could determine its relative efficacy and potential for combination therapies. Moreover, dose-response studies are necessary to optimize therapeutic outcomes while minimizing adverse effects. Exploring functional and behavioral changes, including mobility and overall well-being, would provide a more comprehensive understanding of astaxanthin’s impact. Finally, translational studies in other species, including humans, could establish its broader applicability as a therapeutic agent for diabetes and its complications. These future directions will be crucial for advancing the clinical potential of astaxanthin.

## Conclusions

**Fig 9:**
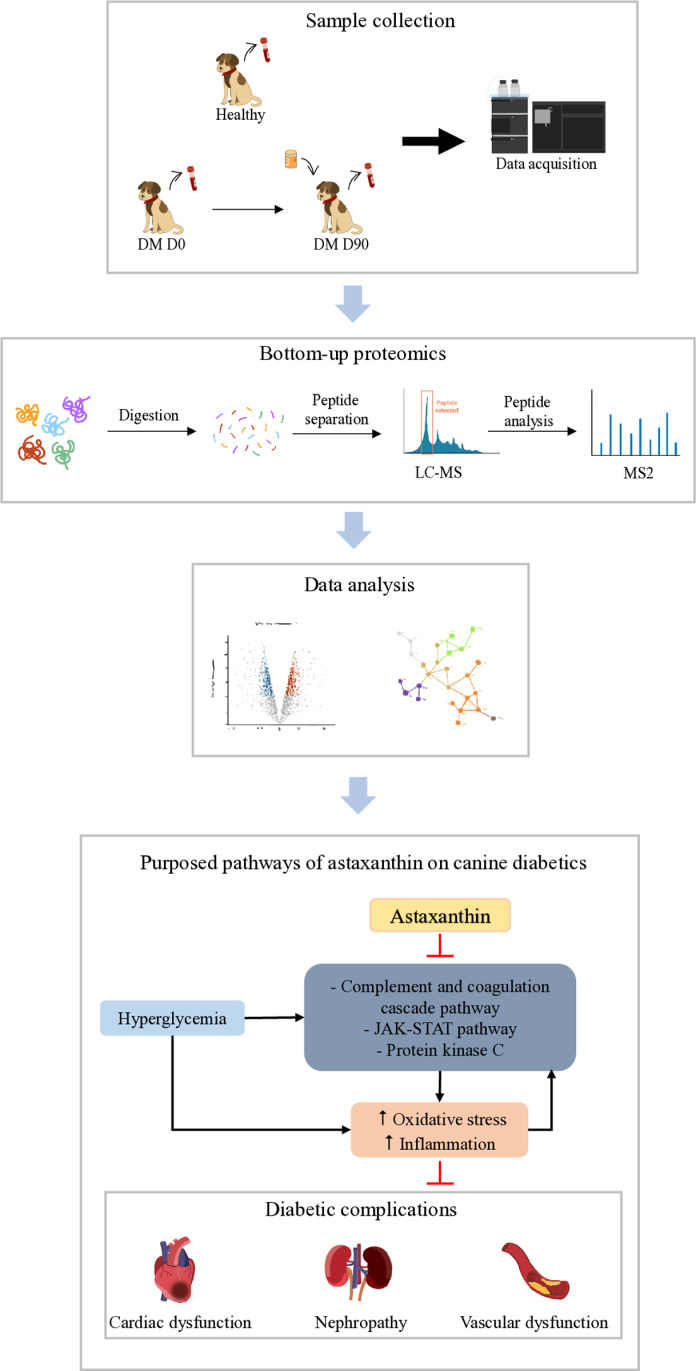
Proteomics pathways involved in diabetes and the effects of astaxanthin supplementation in dogs.

This study provides preliminary insights into the molecular mechanisms of diabetes in canines and the potential effects of astaxanthin supplementation. Astaxanthin modulated critical proteomics pathways associated with inflammation and oxidative stress, such as the complement and coagulation cascades, JAK–STAT signaling, and protein kinase C signaling. These pathways are implicated in the pathogenesis of diabetes. However, the study did not identify changes in clinical parameters, such as blood glucose levels. The proteomics effects observed may reflect early or intermediate responses, suggesting that longer-term or dose-dependent studies may be required to confirm their clinical significance. Fig 9 summarizes the key findings of the proteomics analysis, which focused on proteins that are potentially involved in diabetes and the effects of astaxanthin treatment.

## Supporting information

S1 Table Characteristics of the diabetic dog population (n = 6). (PDF)

S2 Table Clinical parameters of diabetic dogs before and after astaxanthin supplementation. Hematological and blood chemistry parameters (BUN, creatinine, ALT, ALP, AST, glucose, albumin, globulin, calcium, and total bilirubin). NLR, neutrophil to lymphocyte ratio; NMR, neutrophil to monocyte ratio; LMR, lymphocyte to monocyte ratio; PLR, platelet to lymphocyte ratio. *Statistically significant at *P* < 0.05.
(PDF)

S3 Table Upregulated and downregulated proteins in diabetic dogs (DM D0) vs. healthy adult dogs and diabetic dogs after astaxanthin supplementation (DM D90). (PDF)

## References

[1] Shields EJ, Lam CJ, Cox AR, Rankin MM, Van Winkle TJ, Hess RS, et al. Extreme Beta-Cell Deficiency in Pancreata of Dogs with Canine Diabetes. PLOS ONE. 2015;10(6):e0129809. doi: 10.1371/journal.pone.0129809 26057531 PMC4461304

[2] Behrend E, Holford A, Lathan P, Rucinsky R, Schulman R. 2018 AAHA Diabetes Management Guidelines for Dogs and Cats. J Am Anim Hosp Assoc. 2018;54(1):1–21. doi: 10.5326/JAAHA-MS-6822 29314873

[3] Davison LJ, Ristic JM., Herrtage ME, Ramsey IK, Catchpole B. Anti-insulin antibodies in dogs with naturally occurring diabetes mellitus. Veterinary Immunology and Immunopathology, 2003. 91(1):53-60. 12507850 10.1016/s0165-2427(02)00267-2

[4] Papatheodorou K, Banach M, Bekiari E, Rizzo M, Edmonds M. Complications of Diabetes J Diabetes Res 2016 2016:6989453 27822482 10.1155/2016/6989453 PMC5086373

[5] Brownlee M. The pathobiology of diabetic complications: a unifying mechanism. Diabetes. 2005;54(6):1615–25. doi: 10.2337/diabetes.54.6.1615 15919781

[6] Newsholme P, et al Molecular mechanisms of ROS production and oxidative stress in diabetes Biochem J. 2016;473(24):4527–550 27941030 10.1042/BCJ20160503C

[7] Darenskaya MA, Kolesnikova LI, Kolesnikov SI. Oxidative Stress: Pathogenetic Role in Diabetes Mellitus and Its Complications and Therapeutic Approaches to Correction. Bulletin of Experimental Biology and Medicine. 2021;171(2):179–189. doi: 10.1007/s10517-021-05191-7 34173093 PMC8233182

[8] Wong TY, Cheung CMG, Larsen M, Sharma S, Simó R. Diabetic retinopathy. Nat Rev Dis Primers. 2016;2():16012. doi: 10.1038/nrdp.2016.12 27159554

[9] González P, Lozano P, Ros G, Solano F. Hyperglycemia and Oxidative Stress: An Integral, Updated and Critical Overview of Their Metabolic Interconnections. Int J Mol Sci. 2023;24(11):9352. doi: 10.3390/ijms24119352 37298303 PMC10253853

[10] Zamanian M.Y., Alsaab H.O., Golmohammadi M., Yumashev A., Jabba A.M., Abid M.K., et al. NF-κB pathway as a molecular target for curcumin in diabetes mellitus treatment: Focusing on oxidative stress and inflammation. Cell Biochem Funct. 2024;42(4):e4030. doi: 10.1002/cbf.4030 38720663

[11] Leenders F, Groen N, de Graaf N, Engelse MA, Rabelink TJ, de Koning EJP, et al. Oxidative Stress Leads to β-Cell Dysfunction Through Loss of β-Cell Identity. Front Immunol. 2021;12:690379. doi: 10.3389/fimmu.2021.690379 34804002 PMC8601632

[12] Sagoo MK, Gnudi L. Diabetic nephropathy: Is there a role for oxidative stress?. Free Radic Biol Med. 2018;116:50–63. doi: 10.1016/j.freeradbiomed.2017.12.040 29305106

[13] Kowluru RA, Chan P-S. Oxidative stress and diabetic retinopathy. Exp Diabetes Res. 2007;2007:43603. doi: 10.1155/2007/43603 17641741 PMC1880867

[14] Pang L et al Understanding Diabetic Neuropathy: Focus on Oxidative Stress Oxidative Medicine and Cellular Longevity 2020;2020(1)9524635 32832011 10.1155/2020/9524635 PMC7422494

[15] Bosco O, Vizio B, Gruden G, Schiavello M, Lorenzati B, Cavallo-Perin P, et al. Thrombopoietin Contributes to Enhanced Platelet Activation in Patients with Type 1 Diabetes Mellitus. Int J Mol Sci. 2021;22(13):7032. doi: 10.3390/ijms22137032 34210000 PMC8269076

[16] Flyvbjerg A. The role of the complement system in diabetic nephropathy. Nat Rev Nephrol. 2017;13(5):311–318. doi: 10.1038/nrneph.2017.31 28262777

[17] Keaney JF, Loscalzo J. Diabetes, Oxidative Stress, and Platelet Activation. Circulation. 1999 99(2): 189-191. 9892579 10.1161/01.cir.99.2.189

[18] Oguntibeju OO. Type 2 diabetes mellitus, oxidative stress and inflammation: examining the links. Int J Physiol Pathophysiol Pharmacol. 2019;11(3):45–63. 31333808 PMC6628012

[19] Tsalamandris S, et al The Role of Inflammation in Diabetes: Current Concepts and Future Perspectives. European Cardiology Review 2019;14:50 31131037 10.15420/ecr.2018.33.1 PMC6523054

[20] Dludla PV, Mabhida SE, Ziqubu K, Nkambule BB, Mazibuko-Mbeje SE, Hanser S. Pancreatic β-cell dysfunction in type 2 diabetes: Implications of inflammation and oxidative stress. World J Diabetes, 2023. 14(3):130–146. 37035220 10.4239/wjd.v14.i3.130 PMC10075035

[21] van de Vyver M. Immunology of chronic low-grade inflammation: relationship with metabolic function. J Endocrinol. 2023;257(1):e220271. doi: 10.1530/JOE-22-0271 36688876

[22] Donath MY, Shoelson SE. Type 2 diabetes as an inflammatory disease. Nature Reviews Immunology, 2011;11(2):98–107. 10.1038/nri2925 21233852

[23] Chen ZZ, Gerszten RE. Metabolomics and Proteomics in Type 2 Diabetes. Circ Res, 2020. 126(11): p. 1613-1627. 32437301 10.1161/CIRCRESAHA.120.315898 PMC11118076

[24] Yuan S, Xu F, Li X, Chen J, Zheng J, Mantzoros CS, et al. Plasma proteins and onset of type 2 diabetes and diabetic complications: Proteome-wide Mendelian randomization and colocalization analyses. Cell Rep Med. 2023;4(9):101174. doi: 10.1016/j.xcrm.2023.101174 37652020 PMC10518626

[25] Sriboonvorakul N, et al. Proteomics Studies in Gestational Diabetes Mellitus: A Systematic Review and Meta-Analysis J Clin Med 2022 11(10) 10.3390/jcm11102737 PMC9143836 35628864

[26] Zhou T, Huang L, Wang M, Chen D, Chen Z, Jiang S-W. A Critical Review of Proteomic Studies in Gestational Diabetes Mellitus. J Diabetes Res. 2020;2020:6450352. doi: 10.1155/2020/6450352 32724825 PMC7381988

[27] Suemanotham N, Photcharatinnakorn P, Chantong B, Buranasinsup S, Phochantachinda S, Sakcamduang W, et al. Curcuminoid supplementation in canine diabetic mellitus and its complications using proteomic analysis. Front Vet Sci. 2022;9:1057972. doi: 10.3389/fvets.2022.1057972 36619946 PMC9816143

[28] Bungau S.G., et al., Antioxidant and Hypoglycemic Potential of Essential Oils in Diabetes Mellitus and Its Complications. International Journal of Molecular Sciences, 2023. 24(22):16501. 38003691 10.3390/ijms242216501 PMC10671358

[29] Landon R, et al, Impact of Astaxanthin on Diabetes Pathogenesis and Chronic Complications, Marine Drugs. 2020;18(7):357. 32660119 10.3390/md18070357 PMC7401277

[30] Ma B, Lu J, Kang T, Zhu M, Xiong K, Wang J. Astaxanthin supplementation mildly reduced oxidative stress and inflammation biomarkers: a systematic review and meta-analysis of randomized controlled trials. Nutr Res. 2022;99:40–50. doi: 10.1016/j.nutres.2021.09.005 35091276

[31] Fassett RG, Coombes JS. Astaxanthin in cardiovascular health and disease. Molecules. 2012;17(2):2030–48. doi: 10.3390/molecules17022030 22349894 PMC6268807

[32] Wu D., et al. Effects of Astaxanthin Supplementation on Oxidative Stress. Int J Vitam Nutr Res, 2020, 90(1-2): 179–194. 30982442 10.1024/0300-9831/a000497

[33] Medoro A, Intrieri M, Passarella D, Willcox DC, Davinelli S, Scapagnini G. Astaxanthin as a metabolic regulator of glucose and lipid homeostasis. Journal of Functional Foods. 2024;112:105937. doi: 10.1016/j.jff.2023.105937

[34] Murai T, Kawasumi K, Tominaga K, Okada Y, Kobayashi M, Arai T. Effects of astaxanthin supplementation in healthy and obese dogs. Vet Med (Auckl). 2019;10:29–35. doi: 10.2147/VMRR.S186202 30859086 PMC6385744

[35] Chew B.P., Mathison B.D., Hayek M.G., Massimino S., Reinhart G.A., Park J.S. Dietary astaxanthin enhances immune response in dogs. Vet Immunol Immunopathol. 2011;140(3–4):199–206. doi: 10.1016/j.vetimm.2010.12.004 21208664

[36] Zanghi B.M., Middleton R.P., Reynolds A.J. Effects of postexercise feeding of a supplemental carbohydrate and protein bar with or without astaxanthin from Haematococcus pluvialis to exercise-conditioned dogs. Am J Vet Res. 2015;76(4): 338–350. 25815575 10.2460/ajvr.76.4.338

[37] Park JS, Mathison BD, Hayek MG, Zhang J, Reinhart GA, Chew BP. Astaxanthin modulates age-associated mitochondrial dysfunction in healthy dogs. J Anim Sci. 2013;91(1):268–275. doi: 10.2527/jas.2012-5341 23100599

[38] Park JS, Kim HW, Mathison BD, Hayek MG, Massimino S, Reinhart GA. et al., Astaxanthin uptake in domestic dogs and cats. Nutrition & Metabolism, 2010;7(1):52. 20565958 10.1186/1743-7075-7-52 PMC2898833

[39] Perez-Riverol Y, Bai J, Bandla C, García-Seisdedos D, Hewapathirana S, Kamatchinatha S, et al., The PRIDE database resources in 2022: a hub for mass spectrometry-based proteomics evidences. Nucleic Acids Research, 2021. 50(D1):D543–D552. 10.1093/nar/gkab1038 PMC8728295 34723319

[40] Szklarczyk D, Gable AL, Lyon D, Junge A, Wyder S, Huerta-Cepas J, et al., STRING v11: protein–protein association networks with increased coverage, supporting functional discovery in genome-wide experimental datasets. Nucleic Acids Research. 2018. 47(D1):D607-D613. 10.1093/nar/gky1131 PMC6323986 30476243

[41] Giwa AM, Ahmed R, Omidian Z, Majety N, Karakus KE, Omer SM, et al. Current understandings of the pathogenesis of type 1 diabetes: Genetics to environment. World J Diabetes. 2020;11(1):13–25. doi: 10.4239/wjd.v11.i1.13 31938470 PMC6927819

[42] Ajjan R.A., Schroeder V. Role of complement in diabetes. Molecular Immunology. 2019;114:270–277. doi: 10.1016/j.molimm.2019.07.031 31400630

[43] Pechlivani N, Ajjan RA. Thrombosis and Vascular Inflammation in Diabetes: Mechanisms and Potential Therapeutic Targets. Front Cardiovasc Med. 2018;5:1. doi: 10.3389/fcvm.2018.00001 29404341 PMC5780411

[44] Morris R, Kershaw NJ, Babon JJ. The molecular details of cytokine signaling via the JAK/STAT pathway. Protein Sci. 2018;27(12):1984–2009. doi: 10.1002/pro.3519 30267440 PMC6237706

[45] Villarino A.V., Kanno Y, Ferdinand J.R., O’Shea J.J. Mechanisms of Jak/STAT signaling in immunity and disease. J Immunol. 2015;194(1):21–7. doi: 10.4049/jimmunol.1401867 25527793 PMC4524500

[46] Pereira CPM, Souza ACR, Vasconcelos AR, Prado PS Antioxidant and anti‑inflammatory mechanisms of action of astaxanthin in cardiovascular diseases (Review). Int J Mol Med. 2021 47(1):37-48. 10.3892/ijmm.2020.4783 PMC7723678 33155666

[47] Chang MX, Xiong F. Astaxanthin and its Effects in Inflammatory Responses and Inflammation-Associated Diseases: Recent Advances and Future Directions. Molecules. 2020;25(22):5342. doi: 10.3390/molecules25225342 33207669 PMC7696511

[48] Kohandel Z, et al. Anti-inflammatory action of astaxanthin and its use in the treatment of various diseases. Biomed Pharmacother, 2022, 145: 112179. 34736076 10.1016/j.biopha.2021.112179

[49] Ellulu MS, Samouda H. Clinical and biological risk factors associated with inflammation in patients with type 2 diabetes mellitus. BMC Endocrine Disorders, 2022. 22(1):16. 34991564 10.1186/s12902-021-00925-0 PMC8740444

[50] Zeng Y, Li Y, Jiang W, Hou N. Molecular mechanisms of metabolic dysregulation in diabetic cardiomyopathy. Front Cardiovasc Med. 2024;11:1375400. doi: 10.3389/fcvm.2024.1375400 38596692 PMC11003275

[51] Boucher J, Kleinridders A, Kahn CR. Insulin receptor signaling in normal and insulin-resistant states. Cold Spring Harb Perspect Biol. 2014;6(1):a009191. doi: 10.1101/cshperspect.a009191 24384568 PMC3941218

[52] Le TKC, Dao XD, Nguyen DV, Luu DH, Bui TMH, Le TH, et al. Insulin signaling and its application. Front Endocrinol (Lausanne). 2023;14:1226655. doi: 10.3389/fendo.2023.1226655 37664840 PMC10469844

[53] Hameed I, Masoodi SR, Mir SA, Nabi M, Ghazanfar K, Ganai BA. Type 2 diabetes mellitus: From a metabolic disorder to an inflammatory condition. World J Diabetes. 2015;6(4):598–612. doi: 10.4239/wjd.v6.i4.598 25987957 PMC4434080

[54] Dilworth L, Facey A, Omoruyi F. Diabetes Mellitus and Its Metabolic Complications: The Role of Adipose Tissues. Int J Mol Sci. 2021;22(14):7644. doi: 10.3390/ijms22147644 34299261 PMC8305176

[55] Kanwugu O.N., Glukhareva T.V., Danilova I.G., Kovaleva E.G. Natural antioxidants in diabetes treatment and management: prospects of astaxanthin. Crit Rev Food Sci Nutr. 2022;62(18):5005–5028. doi: 10.1080/10408398.2021.1881434 33591215

[56] Feng W, Wang Y, Guo N, Huang P, Mi Y. Effects of Astaxanthin on Inflammation and Insulin Resistance in a Mouse Model of Gestational Diabetes Mellitus. Dose Response. 2020;18(2):1559325820926765. doi: 10.1177/1559325820926765 32501299 PMC7241269

[57] Ghosh P, Sahoo R, Vaidya A, Chorev M, Halperin JA. Role of complement and complement regulatory proteins in the complications of diabetes. Endocr Rev.2015. 36(3):272–88. 25859860 10.1210/er.2014-1099 PMC4446516

[58] King BC, Blom AM. Complement in metabolic disease: metaflammation and a two-edged sword. Seminars in Immunopathology, 2021;43(6):829–841. 34159399 10.1007/s00281-021-00873-w PMC8613079

[59] Shim K, Begum R, Yang C, Wang H. Complement activation in obesity, insulin resistance, and type 2 diabetes mellitus. World J Diabetes. 2020;11(1):1–12. doi: 10.4239/wjd.v11.i1.1 31938469 PMC6927818

[60] Jiang F et al The complement system and diabetic retinopathy. Surv Ophthalmol. 2024 10.1016/j.survophthal.2024.02.004 38401574

[61] Li X, et al. Effects of Hyperglycemia and Diabetes Mellitus on Coagulation and Hemostasis. Journal of Clinical Medicine. 2021;10(11):2419. 34072487 10.3390/jcm10112419 PMC8199251

[62] Ceriello A. Coagulation activation in diabetes mellitus: the role of hyperglycaemia and therapeutic prospects. Diabetologia. 1993;36(11):1119–25. doi: 10.1007/BF00401055 8270125

[63] Safdar NZ, Kietsiriroje N, Ajjan RA. The Cellular and Protein Arms of Coagulation in Diabetes: Established and Potential Targets for the Reduction of Thrombotic Risk. Int J Mol Sci. 2023;24(20):15328. doi: 10.3390/ijms242015328 37895008 PMC10607436

[64] Keindl M, et al Increased Plasma Soluble Interleukin-2 Receptor Alpha Levels in Patients With Long-Term Type 1 Diabetes With Vascular Complications Associated With IL2RA and PTPN2 Gene Polymorphisms Frontiers in Endocrinology. 2020;(11) 10.3389/fendo.2020.575469 PMC7664831 33193091

[65] Watanabe K, Kawamura N, Tokuda M, Oguni T, Konishi K, Mino M. Soluble interleukin-2 receptor level in serum of children with insulin-dependent diabetes mellitus compared with that in diseases with activated immune system. Pathophysiology. 1996;3(1):37–40. doi: 10.1016/0928-4680(95)00048-8

[66] Ader M, Bergman R.N. Hyperinsulinemic Compensation for Insulin Resistance Occurs Independent of Elevated Glycemia in Male Dogs. Endocrinology, 2021;162(9). 10.1210/endocr/bqab119 PMC8282122 34132779

[67] Esser N, Utzschneider KM, Kahn SE. Early beta cell dysfunction vs insulin hypersecretion as the primary event in the pathogenesis of dysglycaemia. Diabetologia. 2020;63(10):2007–2021. doi: 10.1007/s00125-020-05245-x 32894311

[68] Huang X, Liu G, Guo J, Su Z. The PI3K/AKT pathway in obesity and type 2 diabetes Int J Biol Sci 2018;14 11:1483–1496 30263000 10.7150/ijbs.27173 PMC6158718

[69] Ramasubbu K, Devi Rajeswari V. Impairment of insulin signaling pathway PI3K/Akt/mTOR and insulin resistance induced AGEs on diabetes mellitus and neurodegenerative diseases: a perspective review. Mol Cell Biochem. 2023;478(6):1307–1324. doi: 10.1007/s11010-022-04587-x 36308670

[70] Naseri R, Navabi SJ, Samimi Z, Mishra AP, Nigam M, Chandra H, et al. Targeting Glycoproteins as a therapeutic strategy for diabetes mellitus and its complications. Daru. 2020;28(1):333–58. doi: 10.1007/s40199-020-00327-y 32006343 PMC7095136

[71] Pretorius L, Thomson GJA, Adams RCM, Nell TA, Laubscher WA, Pretorius E. Platelet activity and hypercoagulation in type 2 diabetes. Cardiovasc Diabetol. 2018;17(1):141. doi: 10.1186/s12933-018-0783-z 30388964 PMC6214175

[72] Domingueti CP, Dusse LMS, Carvalho M das G, Gomes KB, Fernandes AP. Hypercoagulability and cardiovascular disease in diabetic nephropathy. Clin Chim Acta. 2013;415:279–285. doi: 10.1016/j.cca.2012.10.061 23159842

[73] Verkleij CJ, Roelofs JJ, Havik SR, Meijers JC, Marx PF. The role of thrombin-activatable fibrinolysis inhibitor in diabetic wound healing. Thromb Res. 2010. 126(5):442–6. 20828799 10.1016/j.thromres.2010.08.008

[74] Guo H, Yan Z, Hu Y, Huang X, Pan C. Complement C7 is Specifically Expressed in Mesangial Cells and is a Potential Diagnostic Biomarker for Diabetic Nephropathy and is Regulated by miR-494-3p and miR-574-5p. Diabetes Metab Syndr Obes. 2021;14:3077–88. doi: 10.2147/DMSO.S311725 34262312 PMC8273746

[75] Sircar M, Rosales IA, Selig MK, Xu D, Zsengeller ZK, Stillman IE, et al. Complement 7 Is Up-Regulated in Human Early Diabetic Kidney Disease. Am J Pathol. 2018;188(10):2147–2154. doi: 10.1016/j.ajpath.2018.06.018 30253844 PMC6180251

[76] van Greevenbroek M.M., Ghosh S, van der Kallen C.J.H., Brouwers M.C.G.J., Schalkwijk C.G., Stehouwer C.D.A. Up-regulation of the complement system in subcutaneous adipocytes from nonobese, hypertriglyceridemic subjects is associated with adipocyte insulin resistance. J Clin Endocrinol Metab. 2012;97(12):4742–52. doi: 10.1210/jc.2012-2539 23055543 PMC3513546

[77] Liu S-L, Wu NQ, Shi HW, Dong Q, Dong QT, Gao Y, et al. Fibrinogen is associated with glucose metabolism and cardiovascular outcomes in patients with coronary artery disease. Cardiovascular Diabetology, 2020. 19(1):36. 32192491 10.1186/s12933-020-01012-9 PMC7081587

[78] Raynaud E, Pérez-Martin A, Brun JF, Aïssa-Benhaddad A, Fédou C, Mercier J. Relationships between fibrinogen and insulin resistance. Atherosclerosis, 2000. 150(2):365–70. 10856528 10.1016/s0021-9150(99)00373-1

[79] Carr ME. Diabetes mellitus: A hypercoagulable state. Journal of Diabetes and its Complications, 2001. 15(1):44–54. 11259926 10.1016/s1056-8727(00)00132-x

[80] Edén D, Panagiotou G, Mokhtari D, Eriksson JW, Åberg M, Siegbahn A. Adipocytes express tissue factor and FVII and are procoagulant in a TF/FVIIa-dependent manner. Upsala Journal of Medical Sciences. 2019;124(3):158–167. doi: 10.1080/03009734.2019.1645248 31407948 PMC6758637

[81] Pap D, Veres-Székely A, Szebeni B, Rokonay R, Ónody A, Lippai R, et al. Characterization of IL-19, -20, and -24 in acute and chronic kidney diseases reveals a pro-fibrotic role of IL-24. Journal of Translational Medicine. 2020;18(1):172. doi: 10.1186/s12967-020-02338-4 32306980 PMC7168946

[82] Boniface K., et al., From interleukin-23 to T-helper 17 cells: human T-helper cell differentiation revisited. Immunological Reviews, 2008. 226(1):132–146. 19161421 10.1111/j.1600-065X.2008.00714.x PMC3660846

[83] Fatima N, et al, Emerging role of Interleukins IL-23/IL-17 axis and biochemical markers in the pathogenesis of Type 2 Diabetes: Association with age and gender in human subjects, Int J Biol Macromol, 2017. 105 Pt 1: 1279–1288. 28757426 10.1016/j.ijbiomac.2017.07.155

[84] Lien C.-F., et al. Potential Role of Protein Kinase C in the Pathophysiology of Diabetes-Associated Atherosclerosis Frontiers in Pharmacology 2021;12 10.3389/fphar.2021.716332 PMC8283198 34276388

[85] Pan D, Xu L, Guo M. The role of protein kinase C in diabetic microvascular complications. Front Endocrinol (Lausanne). 2022;13:973058. doi: 10.3389/fendo.2022.973058 36060954 PMC9433088

[86] Geraldes P, King GL. Activation of protein kinase C isoforms and its impact on diabetic complications. Circ Res, 2010. 106(8):1319–31. 20431074 10.1161/CIRCRESAHA.110.217117 PMC2877591

[87] Hodgkinson C.P., Mander A., Sale G.J. Identification of 80K-H as a protein involved in GLUT4 vesicle trafficking. Biochem J. 2005;388(Pt 3):785–93. doi: 10.1042/BJ20041845 15707389 PMC1183457

[88] Herget T, Brooks SF, Broad S, Rozengurt E. Relationship between the major protein kinase C substrates acidic 80-kDa protein-kinase-C substrate (80K) and myristoylated alanine-rich C-kinase substrate (MARCKS). European Journal of Biochemistry. 1992;209(1):7–14. doi: 10.1111/j.1432-1033.1992.tb17255.x 1396720

[89] Hu X, Li J, Fu M, Zhao X, Wang W. The JAK/STAT signaling pathway: from bench to clinic. Signal Transduct Target Ther. 2021;6(1):402. doi: 10.1038/s41392-021-00791-1 34824210 PMC8617206

[90] Wei J, Yuan Y, Jin C, Chen H, Leng L, He F, et al. The ubiquitin ligase TRAF6 negatively regulates the JAK-STAT signaling pathway by binding to STAT3 and mediating its ubiquitination. PLOS ONE. 2012;7(11):e49567. doi: 10.1371/journal.pone.0049567 23185365 PMC3501508

[91] Hatakeyama S. Ubiquitin-mediated regulation of JAK-STAT signaling in embryonic stem cells. JAKSTAT. 2012;1(3):168–75. doi: 10.4161/jkst.21560 24058766 PMC3670240

[92] Guo W, Tian D, Jia Y, Huang W, Jiang M, Wang J, et al. MDM2 controls NRF2 antioxidant activity in prevention of diabetic kidney disease. Biochim Biophys Acta Mol Cell Res. 2018;1865(8):1034–1045. doi: 10.1016/j.bbamcr.2018.04.011 29704532

[93] Davinelli S, Saso L, D’Angeli F, Calabrese V, Intrieri M, Scapagnini G. Astaxanthin as a Modulator of Nrf2, NF-κB, and Their Crosstalk: Molecular Mechanisms and Possible Clinical Applications. Molecules. 2022;27(2):502. doi: 10.3390/molecules27020502 35056816 PMC8779084

[94] Kowshik J, Baba AB, Giri H, Deepak Reddy G, Dixit M, Nagini S. Astaxanthin inhibits JAK/STAT-3 signaling to abrogate cell proliferation, invasion and angiogenesis in a hamster model of oral cancer. PLoS One. 2014;9(10):e109114. doi: 10.1371/journal.pone.0109114 25296162 PMC4189964

[95] Zarneshan S.N., Fakhri S., Farzaei M.H., Khan H., Saso L. Astaxanthin targets PI3K/Akt signaling pathway toward potential therapeutic applications. Food Chem Toxicol. 2020;145:111714. doi: 10.1016/j.fct.2020.111714 32871194

[96] Deng Z-Y, Shan W-G, Wang S-F, Hu M-M, Chen Y. Effects of astaxanthin on blood coagulation, fibrinolysis and platelet aggregation in hyperlipidemic rats. Pharm Biol. 2017;55(1):663–672. doi: 10.1080/13880209.2016.1261905 27951728 PMC6130668

[97] Lauver D.A., Lockwood S.F., Lucchesi B.R. Disodium Disuccinate Astaxanthin (Cardax) attenuates complement activation and reduces myocardial injury following ischemia/reperfusion. J Pharmacol Exp Ther, 2005. 314(2): 686–92. 15872041 10.1124/jpet.105.087114

[98] Papachristoforou E, Lambadiari V, Maratou E, Makrilakis K. Association of Glycemic Indices (Hyperglycemia, Glucose Variability, and Hypoglycemia) with Oxidative Stress and Diabetic Complications. J Diabetes Res. 2020;2020:7489795. doi: 10.1155/2020/7489795 33123598 PMC7585656

[99] Dandona P, Ghanim H, Chaudhuri A, Dhindsa S, Kim SS. Macronutrient intake induces oxidative and inflammatory stress: potential relevance to atherosclerosis and insulin resistance. Exp Mol Med. 2010;42(4):245–53. doi: 10.3858/emm.2010.42.4.033 20200475 PMC2859324

[100] Shiel R.E., Mooney C.T. Insulins for the long term management of diabetes mellitus in dogs: a review. Canine Med Genet. 2022;9(1):1. doi: 10.1186/s40575-022-00114-9 35152907 PMC8842735

[101] Pellegrini V, La Grotta R, Carreras F, Giuliani A, Sabbatinelli J, Olivieri F, et al. Inflammatory Trajectory of Type 2 Diabetes: Novel Opportunities for Early and Late Treatment. Cells. 2024;13(19):1662. doi: 10.3390/cells13191662 39404426 PMC11476093

[102] Pradhan A.D., Manson J.E., Rifai N., Buring J.E., Ridker P.M. C-reactive protein, interleukin 6, and risk of developing type 2 diabetes mellitus. JAMA. 2001;286(3):327–34. doi: 10.1001/jama.286.3.327 11466099

[103] Mann M. Can proteomics retire the western blot?. J Proteome Res. 2008;7(8):3065. doi: 10.1021/pr800463v 18671370

[104] Baker M. Reproducibility crisis: Blame it on the antibodies. Nature. 2015;521(7552):274–6. doi: 10.1038/521274a 25993940

[105] Blein-Nicolas M, Zivy M. Thousand and one ways to quantify and compare protein abundances in label-free bottom-up proteomics. Biochim Biophys Acta. 2016;1864(8):883–895. doi: 10.1016/j.bbapap.2016.02.019 26947242

[106] Vogel C, Marcotte EM. Calculating absolute and relative protein abundance from mass spectrometry-based protein expression data. Nat Protoc. 2008;3(9):1444–1451. doi: 10.1038/nprot.2008.132 18772871

[107] Silva JC, Denny R, Dorschel CA, Gorenstein M, Kass IJ, Li G-Z, et al. Quantitative proteomic analysis by accurate mass retention time pairs. Anal Chem. 2005;77(7):2187–200. doi: 10.1021/ac048455k 15801753

[108] Aebersold R, Mann M. Mass spectrometry-based proteomics. Nature. 2003;422(6928):198–207. doi: 10.1038/nature01511 12634793

[109] Aebersold R, Mann M. Mass-spectrometric exploration of proteome structure and function. Nature. 2016;537(7620):347–55. doi: 10.1038/nature19949 27629641

