# Supplementary material for: Plasma-based proteomics analysis of molecular pathways in canine diabetes mellitus after astaxanthin supplementation
Source: PLoS One. 2025 May 7;20(5):e0321509. doi: 10.1371/journal.pone.0321509 (PMC12057883; doi:10.1371/journal.pone.0321509)
Supplement: S1 Table — (PDF) [file pone.0321509.s001.pdf]

Supporting information 1

Supporting table 1. Population characteristic of diabetic dogs (n = 6).

DM Dog No	Sex	Age (year)	Breed	BW (Kg)	BCS
1	F	5	Mixed	10	4/9
2	F	9	Shih Tzu	6.5	5/9
3	M	6	Mixed	7.5	4/9
4	M	7	Chihuahua	5	5/9
5	F	11	Chihuahua	5.5	5/9
6	M	12	American cocker spaniel	9.6	4/9
