# Supplementary material for: Plasma-based proteomics analysis of molecular pathways in canine diabetes mellitus after astaxanthin supplementation
Source: PLoS One. 2025 May 7;20(5):e0321509. doi: 10.1371/journal.pone.0321509 (PMC12057883; doi:10.1371/journal.pone.0321509)
Supplement: S2 Table — Hematological and blood chemistry parameters (BUN, creatinine, ALT, ALP, AST, glucose, albumin, globulin, calcium, and total bilirubin). NLR, neutrophil to lymphocyte ratio; NMR, neutrophil to monocyte ratio; LMR, lymphocyte to monocyte ratio; PLR, platelet to lymphocyte ratio. *Statistically significant at P < 0.05. (PDF) [file pone.0321509.s002.pdf]

Supporting information 2

Quantitative variables were summarized as mean and standard deviation (SD). Difference between study groups were assessed by pair t-test and Wilcoxon rank-sum. Statistical analyses were performed using SPSS Statistics for Windows, version 18.0 (SPSS Inc., Chicago, Ill., USA).

Supporting table 2. Clinical parameters of diabetic dogs before and after astaxanthin supplementation. Hematological and blood chemistry parameters (BUN, creatinine, ALT, ALP, AST, glucose, albumin, globulin, calcium, total bilirubin). NLR = neutrophil to lymphocyte ratio, NMR = neutrophil to monocyte ratio, LMR = lymphocyte to monocyte ratio, PLR = platelet to lymphocyte ratio. *Statistically significant at $p < 0.05$

Parameter	Reference range	Mean (±SD)		p-value
		DM day 0	DM day 90	
WBC	6000-17000	9950 (±3445)	9972 (±2415)	0.975
Monocyte	180-1700	408 (±172)	678 (±313)	0.131
Neutrophil	3600-13090	7607 (±2801)	7250 (±1818)	0.648
Lymphocyte	720-5100	1856 (±635)	1890 (±537)	0.866
Eosinophil	120-1700	79 (±134)	154 (±190)	0.586
Basophil	0-170	0	0	0
RBC	5-9	6.98 (±0.68)	7.01 (±0.594)	0.873
Platelet	200-500	274.8 (±45.207)	303.2 (±89.806)	0.306
NLR		4.23 (±1.029)	3.92 (±0.743)	0.626
NMR		20.72 (±7.677)	11.44 (±2.428)	0.085
LMR		4.98 (±1.747)	3.03 (±1.059)	0.047*
PLR		169.8 (±85.91)	142.61 (±113.928)	0.494

Parameter	Reference range	Mean (±SD)		p-value
		DM day 0	DM day 90	
BUN (mg/dL)	7-27	15.80 (±7.12)	16.20 (±5.97)	0.688
Creatinine (mg/dL)	0.50-1.80	0.54 (±0.11)	0.44 (±0.05)	0.102
ALT (U/L)	10-118	87.40 (±45.50)	57.00 (±17.65)	0.138
ALP (U/L)	20-150	167.60 (±73.82)	31.60 (±5.41)	0.208
AST (U/L)	14-45	38.00 (±8.06)	31.60 (±5.41)	0.098
Glucose (mg/dL)	77-125	270.40 (±89.4)	333.00 (±51.09)	0.251
Albumin (g/dL)	2.7-3.8	3.92 (±0.40)	3.84 (±0.34)	0.099
Globulin (g/dL)	2.5-4.4	2.68 (±0.14)	2.50 (±0.15)	0.144
Total protein	5.2-8.2	6.60 (±0.14)	6.34 (±0.25)	0.033*
Calcium (mg/dL)	7.9-12.0	9.94 (±0.43)	9.92 (±0.57)	0.890
Total bilirubin (mg/dL)	0-0.9	0.30 (±0.07)	0.32 (±0.04)	0.564
