# Supplementary material for: Plasma-based proteomics analysis of molecular pathways in canine diabetes mellitus after astaxanthin supplementation
Source: PLoS One. 2025 May 7;20(5):e0321509. doi: 10.1371/journal.pone.0321509 (PMC12057883; doi:10.1371/journal.pone.0321509)
Supplement: S3 Table — (PDF) [file pone.0321509.s003.pdf]

Supporting information 3

Supporting table 3. Up- and down-regulated proteins in diabetic dogs (DM D0) versus healthy adult dogs and diabetic dogs after astaxanthin supplementation (DM D90).

	Protein ID	Protein name	Q-value	Gene name	Group	Average intensity ± (SD) (×10^{6})	95% CI (lower-upper) (×10^{6})	Function	p-value
Complement and coagulation cascade	A0A8I3PKT4	Complement C1S	0.039	C1S	Healthy adult	0.575 ± (0.380)	0.176-0.974	Component of the classical pathway of the complement system	<0.001
					DM D0	19.205 ± (5.358)	5.900-32.515		
					DM D90	7.051 ± (4.030)	-2.961-17.063		
	A0A8P0T2K7	Complement C7	0.049	C7	Healthy adult	0	0	Constituent of the membrane attack complex (MAC)	<0.001
					DM D0	0.020 ± (0.001)	-0.005-0.044		
					DM D90	0	0		
	A0A8C0MTI9	Fibrinogen alpha chain	0	FGA	Healthy adult	0.294 ± (0.214)	0.069-0.519	One of the primary components of blood clots in hemostasis	<0.001
					DM D0	0.490 ± (0.086)	0.278-0.704		
					DM D90	0	0		
	A0A8I3N8Y1	Fibrinogen gamma chain	0.027	FGG	Healthy adult	0.328 ± (0.130)	0.191-0.464	One of the primary components of blood clots in hemostasis	<0.0001
					DM D0	0.481 ± (0.104)	0.222-0.739		
					DM D90	0	0		
	A0A8P0PC28	Coagulation factor III, tissue factor	0.048	F3	Healthy adult	0	0	Initiates coagulation by forming a complex with circulating factor VII	<0.001
					DM D0	0.004 ± (0.002)	-0.001-0.008		
					DM D90	0	0		
	A0A8C0S477	Coagulation factor X	0.049	F7	Healthy adult	0	0	Converts prothrombin to thrombin	<0.0001
					DM D0	0.402 ± (0.095)	0.165-0.639		
					DM D90	0.026 ± (0.013)	-0.007-0.059		
	A0A8I3NR05	Coagulation factor XII	0.048	F12	Healthy adult	0.229 ± (0.099)	0.125-0.333	Initiates coagulation, fibrinolysis and the generation of	<0.0001
					DM D0	0.450 ± (0.082)	0.295-0.705		

	Protein ID	Protein name	Q-value	Gene name	Group	Average intensity ± (SD) (×10[6])	95% CI (lower-upper) (×10[6])	Function	p-value
					DM D90	0.107 ± (0.011)	0.079-0.136	bradykinin and angiotensin	
**JAK-STAT signaling pathway**	A0A8C0MUJ3	Interleukin-2 receptor subunit beta	0.049	IL2RB	Healthy adult	0	0	Involved in receptor-mediated endocytosis and transduction of the mitogenic signals of IL2	<0.001
					DM D0	0.399 ± (0.174)	-0.034-0.832		
					DM D90	0	0		
	A0A8I3PTK1	Interleukin-20 receptor subunit beta	0.049	IL20RB	Healthy adult	0	0	IL20RA/IL20RB dimer is a receptor for IL19, IL20, and IL24	<0.0001
					DM D0	0.285 ± (0.110)	0.011-0.559		
					DM D90	0	0		
	A0A8I3N7Q4	Interleukin-23 subunit alpha	0.033	IL23A	Healthy adult	0	0	Activation of several pathways including p38 MAPK and NF-kappa-B; promotes the production of proinflammatory cytokines	<0.001
					DM D0	0.034 ± (0.018)	-0.012-0.080		
					DM D90	0	0		
**Insulin secretion**	A0A8P0TL75	Protein kinase C alpha	0.047	PRKCA	Healthy adult	0	0	Stimulates platelets and leads to platelet aggregation	<0.0001
					DM D0	0.051 ± (0.006)	0.037-0.066		
					DM D90	0	0		
**PI3K-Akt signaling pathway**	A0A8I3P0F4	E3 ubiquitin-protein ligase Mdm2	0.049	MDM2	Healthy adult	2.238 ± (0.285)	1.939-2.537	Mediates ubiquitination of p53/TP53, leading to its degradation by the proteasome	<0.0001
					DM D0	0	0		
					DM D90	0.043 ± (0.008)	0.022-0.064		
**Glycoprotein metabolic process**	A0A8P0PKC6	Protein kinase C substrate 80K-H	0.049	PRKCSH	Healthy adult	1.231 ± (0.221)	0.999-1.463	Involved in calcium ion binding and kinase activity	<0.0001
					DM D0	0.029 ± (0.014)	-5702-64318		
					DM D90	1.001 ± (0.144)	0.644-1.358		
